# Omental metastasis as a predictive risk factor for unfavorable prognosis in patients with stage III–IV epithelial ovarian cancer

**DOI:** 10.1007/s10147-021-01866-3

**Published:** 2021-01-29

**Authors:** Yutaka Iwagoi, Takeshi Motohara, Sangyoon Hwang, Koichi Fujimoto, Tokunori Ikeda, Hidetaka Katabuchi

**Affiliations:** 1 Department of Obstetrics and Gynecology, Faculty of Life Sciences, Kumamoto University, 1-1-1 Honjo, Chuo-ku, Kumamoto, Kumamoto 860-8556 Japan; 2 Department of Clinical Laboratory, Fukuoka University Hospital, 7-45-1, Nanakuma, Jonan-ku, Fukuoka, Fukuoka 814-0180 Japan; 3 Laboratory of Clinical Pharmacology and Therapeutics, Faculty of Pharmaceutical Sciences, Sojo University, 4-22-1, Ikeda, Nishi-ku, Kumamoto, Kumamoto 860-0082 Japan; 4 Department of Medical Information Sciences and Administration Planning, Kumamoto University Hospital, 1-1-1 Honjo, Chuo-ku, Kumamoto, Kumamoto 860-8556 Japan

**Keywords:** Ovarian cancer, Omental metastasis, Prognosis, Chemoresistance

## Abstract

**Background:**

Epithelial ovarian cancer has a clear predilection for the omentum as the site of metastasis; however, its contribution to clinical outcomes remains unresolved. This study aimed to evaluate the prognostic significance and efficacy of chemotherapy in the presence of omental metastasis.

**Methods:**

A retrospective cohort study was performed in 56 patients with stage III–IV ovarian cancer who underwent primary debulking surgery between 2004 and 2018 at Kumamoto University Hospital.

**Results:**

Thirty-six (64.3%) patients were categorized into the omental metastasis-positive group, whereas 20 (35.7%) patients were in the omental metastasis-negative group. The 5-year overall survival rates were 43.4% in the omental metastasis-positive group and 93.8% in the omental metastasis-negative group. Statistically significant differences were observed in overall survival (*p* = 0.002) and progression-free survival (*p* = 0.036) between the omental metastasis-positive and metastasis-negative groups. Notably, multivariate analysis demonstrated that the existence of omental metastasis is an independent risk factor for overall survival in patients with stage III–IV ovarian cancer (hazard ratio 8.90, 95% confidence interval 1.16–69.77; *p* = 0.038). Furthermore, the omental metastasis-positive group had significantly lower overall response rates to chemotherapy for recurrent disease, compared to the omental metastasis-negative group (31.6% vs. 85.7%, *p* = 0.026).

**Conclusion:**

Our present data demonstrated that omental metastasis is closely associated with an unfavorable prognosis due to increased chemoresistance in patients with stage III–IV ovarian cancer. Elucidating the biological mechanism of omental metastasis will shed light on novel therapeutic approaches for the management of advanced ovarian cancer patients.

**Supplementary Information:**

The online version contains supplementary material available at 10.1007/s10147-021-01866-3.

## Introduction

Epithelial ovarian cancer has a high metastatic potential and is the leading cause of death from gynecologic malignancy [1, 2]. Effective screening methods to detect it at an early stage are lacking; therefore, most patients with ovarian cancer are diagnosed after the tumor has metastasized to the peritoneum outside the pelvis, such as the omentum, small intestine, mesentery, diaphragm, hepatic surface, and/or to the retroperitoneal lymph nodes (stage III), or metastasized to distant organs, such as liver, bone, spleen, lung, and lymph nodes outside of the abdominal cavity (stage IV) [3]. Owing to the early onset metastasis in the peritoneal cavity, a complete resection in debulking surgery is difficult for patients with advanced ovarian cancer to undergo. Furthermore, many patients with advanced ovarian cancer initially respond to chemotherapy; however, chemoresistant residual tumors can survive in metastatic sites and lead to recurrence [4, 5]. Despite advances in surgical techniques and intensive combination chemotherapy, survival outcomes in patients with advanced ovarian cancer remain unfavorable [6].

Up until the present, previous basic studies and clinical observation have highlighted the fact that ovarian cancer has a clear predilection for metastasis to the omentum in the abdominal cavity [7–9]. Hence, omentectomy has become a standard procedure in primary debulking surgery for all cases of ovarian cancer, despite the absence of a visible tumor in the omentum [10–14]. In addition, a recent study by Böhm et al. demonstrated a relationship between the response of omental metastatic tumors to chemotherapy and the survival outcomes of advanced ovarian cancer patients [15]. Intriguingly, patients with a poor response to chemotherapy for omental metastatic tumors had significantly worse survival outcomes than patients with a good response to chemotherapy in advanced-stage ovarian cancer. These findings suggest that the tumor microenvironment of the omentum has the potential to significantly impact the biological features and clinical outcomes of advanced ovarian cancer.

Although most advanced ovarian cancer patients have omental metastasis, gynecologists occasionally experience patients without omental metastasis, despite being diagnosed with stage III–IV ovarian cancer. However, it remains unclear about the difference of the biological and clinical relevance between the presence and absence of omental metastasis in advanced ovarian cancer patients.

The aim of the current study was to investigate the effect of omental metastasis on the clinicopathological characteristics and survival outcomes of patients with stage III–IV ovarian cancer, based on long-term follow-up.

## Patients and methods

### Patient selection and data collection

**Fig. 1:**
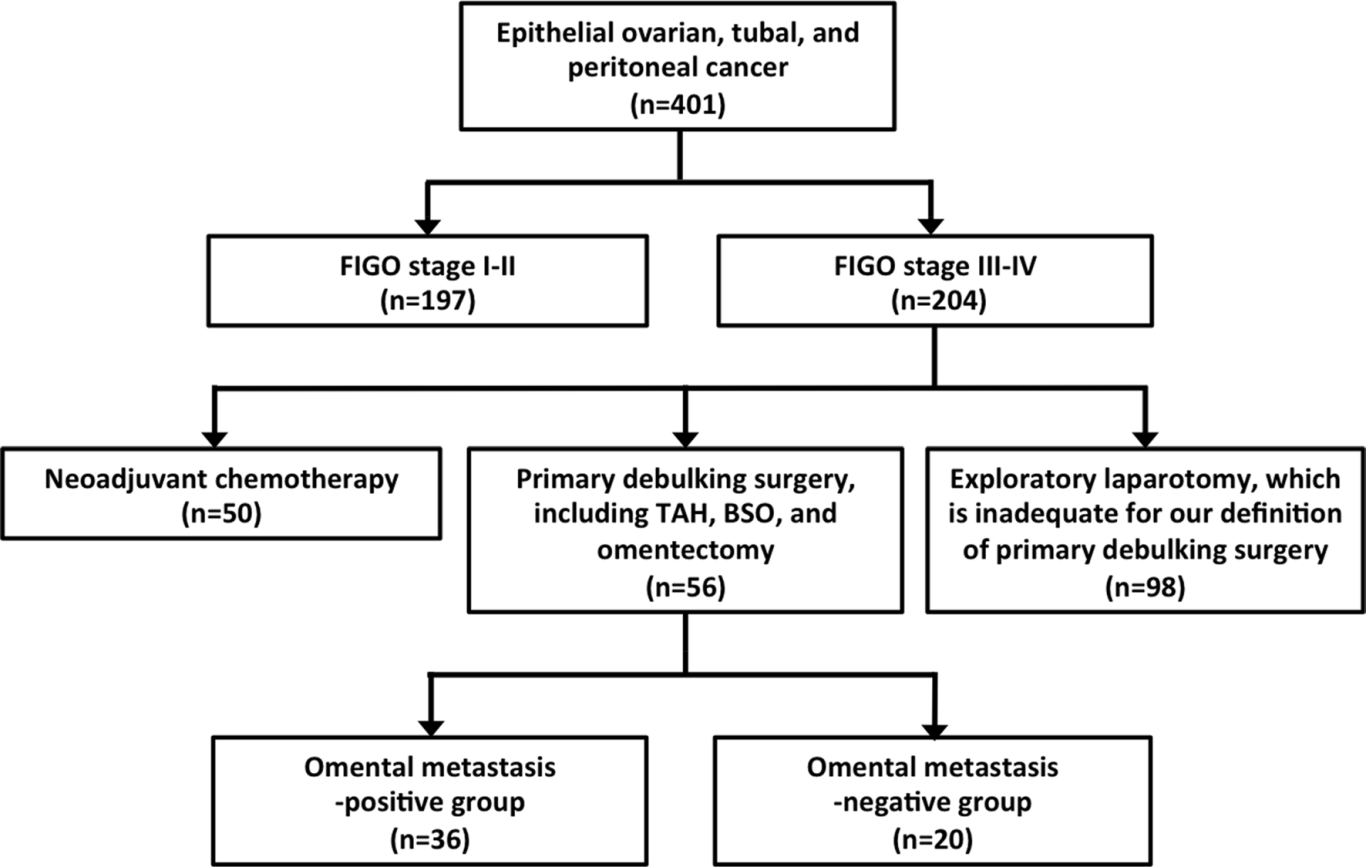
Flowchart of the study design and inclusion of patients. Patients included in our analysis were evaluated for the effect of omental metastasis on clinicopathological characteristics and clinical outcomes. *FIGO* International Federation of Gynecology and Obstetrics, *TAH* total abdominal hysterectomy, *BSO* bilateral salpingo-oophorectomy

We reviewed the medical records of patients with epithelial ovarian, tubal, and peritoneal cancer who were treated at Kumamoto University Hospital (Kumamoto, Japan) from January 2004 to December 2018. Overall, 401 patients received their initial treatment at our institution and were followed up until December 2019. During the study period, patients with stage III–IV ovarian cancer who underwent primary debulking surgery, which included (as a minimum) total abdominal hysterectomy, bilateral salpingo-oophorectomy, and omentectomy, were included in our cohort. With regard to the procedure of omentectomy, we detached the omentum just below the gastroepiploic vessels and excised at the level of transverse colon for all eligible patients. Patients were excluded when they received neoadjuvant chemotherapy or exploratory laparotomy, which was inadequate for our definition of primary debulking surgery (Fig. 1). All excised tissues were examined by experienced pathologists according to the World Health Organization (WHO) classification. Patients were staged in accordance with the International Federation of Gynecology and Obstetrics (FIGO) 2014 ovarian cancer staging system. Based on the results of the histopathological diagnosis, the patients with histologically identified omental metastasis were categorized into the omental metastasis-positive” group, whereas patients without omental metastasis into “the omental metastasis-negative” group. This study was approved by the institutional review board. Written informed consent was obtained from all patients before treatment, based on the institutional guidelines of our hospital.

We defined overall survival (OS) as the date of primary debulking surgery until death or last follow-up. Progression-free survival (PFS) was measured from the date of primary debulking surgery until the first evidence of disease recurrence. Post-recurrence survival (PRS) was defined as the interval from documented recurrence to the time of death or last follow-up.

Treatment response to chemotherapy was evaluated by gynecological examination and radiological assessment. Responses were categorized as a complete response (CR), partial response (PR), stable disease, or progressive disease, based on the WHO Response Evaluation Criteria in Solid Tumors (RECIST 1.1) criteria.

### Statistical analysis

This study was an observational cross-sectional study. Fisher’s exact test and Mann–Whitney *U* test were used to compare the association of clinical factors as categorical variables or as continuous variables, respectively. The survival curves of OS, PFS, and PRS were estimated using the Kaplan–Meier method. Log-rank tests were conducted to compare the two groups. Univariate Cox proportional hazard analyses using age, menopausal status, BMI, histological type, primary site, FIGO stage, tumor size, residual tumor size, CA125 level, volume of ascites, and omental metastasis were employed to estimate the hazard ratio (HR) and 95% confidence interval (CI). Multivariable Cox proportional hazard analysis was also conducted. Covariate selection was determined based on the results of univariate Cox proportional hazard analyses. Schoenfeld residuals were assessed to evaluate the proportional hazards in these models. The statistical analyses were conducted using R software, version 3.6.2 (R Foundation for Statistical Computing, Vienna, Austria). A value of *p* < 0.05 was significant.

## Results

### Characteristics of eligible patients

During the study period, the existence of omental metastasis was evaluated by the assessment of histopathological diagnosis using excised omental tissues from 56 patients who underwent primary debulking surgery, which included total abdominal hysterectomy, bilateral salpingo-oophorectomy, and omentectomy. As a consequence, 36 (64.3%) patients were in the omental metastasis-positive group and 20 (35.7%) patients were in the omental metastasis-negative group among patients with stage III–IV ovarian cancer (Fig. 1).

The association between the clinicopathological characteristics and omental metastasis in 56 patients is shown in Table 1. Clinicopathological features, such as age, menopausal status, BMI, histological type, and primary site did not significantly differ between the omental metastasis-positive and metastasis-negative groups. In addition, no significant differences were observed in FIGO stage, CA125 level, amount of ascites, tumor size, and residual tumor size between the two groups. Adjuvant systematic chemotherapy was administered as clinically indicated in accordance with standard practices, and nearly all patients (55/56, 98.2%) received platinum-based chemotherapy as the first-line chemotherapy. No significant differences were observed in the distribution of the number of cycles of chemotherapy between the two groups (Table 1).

**Table 1:** Relationship between clinicopathologic features and omental metastasis in eligible patients with stage III–IV ovarian cancer

Patient characteristics	Total (*n* = 56)	Omental metastasis		*p* value
		Positive (*n* = 36)	Negative (*n* = 20)	
Median age, years (range)	55.5 (32–80)	56.0 (34–80)	52.0 (32–75)	0.34
Menopausal status (%)				
Pre	21 (37.6)	12 (33.3)	9 (45.0)	
Post	35 (62.5)	24 (66.7)	11 (55.0)	0.41
BMI, *n* (%)				
< 18.5 kg/m^2^	5 (8.3)	3 (8.3)	2 (10.0)	1.00
≥ 18.5 kg/m^2^ and < 25 kg/m^2^	39 (69.6)	24 (66.7)	15 (75.0)	0.56
≥ 25 kg/m^2^	12 (21.4)	9 (25.0)	3 (15.0)	0.51
Histological type, *n* (%)				
High-grade serous	38 (67.9)	24 (66.7)	14 (70.0)	1.00
Low-grade serous	2 (3.6)	2 (5.6)	0 (0)	0.53
Clear cell	6 (10.7)	3 (8.3)	3 (15.0)	0.66
Endometrioid	4 (7.1)	2 (5.6)	2 (10.0)	0.61
Mucinous	1 (1.8)	1 (2.8)	0 (0)	1.00
Others	5 (8.9)	4 (11.1)	1 (5.0)	0.65
Primary site, *n* (%)				
Ovary	47 (83.9)	31 (86.1)	16 (80.0)	
Tube	9 (16.1)	5 (13.9)	4 (20.0)	0.71
Peritoneum	0 (0.0)	0 (0.0)	0 (0)	
FIGO stage, *n* (%)				
III	37 (66.1)	22 (61.1)	15 (75.0)	
IV	19 (33.9)	14 (38.9)	5 (25.0)	0.38
CA125, *n* (%)				
< 500 U/mL	25 (44.6)	13 (36.1)	11 (55.0)	
≥ 500 U/mL	31 (55.4)	23 (63.9)	9 (45.0)	0.26
Ascites, *n* (%)				
< 500 mL	38 (67.9)	21 (63.9)	15 (75.0)	
≥ 500 mL	18 (32.1)	15 (36.1)	5 (25.0)	0.26
Tumor size, *n* (%)				
< 10 cm	29 (51.8)	20 (55.6)	8 (45.0)	
≥ 10 cm	27 (48.2)	16 (44.4)	12 (55.0)	0.40
Residual tumor size, *n* (%)				
< 10 mm (optimal surgery)	35 (62.5)	19 (52.8)	14 (70.0)	
≥ 10 mm (suboptimal surgery)	21 (37.5)	17 (47.2)	6 (30.0)	0.26
First-line chemotherapy, *n* (%)				
Platinum-based chemotherapy	55 (98.2)	35 (97.2)	20 (100.0)	
No adjuvant chemotherapy	1 (1.8)	1 (2.8)	0 (0.0)	1.00
No. of cycles of chemotherapy, *n* (%)				
< 2	40 (71.4)	25 (69.4)	15 (75.0)	
≥ 3	16 (28.6)	11 (30.6)	5 (25.0)	0.76

*BMI* body mass index, *FIGO* International Federation of Gynecology and Obstetrics

### Distribution of metastatic sites

The detailed distribution of metastatic sites at the time of primary debulking surgery is shown in Table 2. Metastatic sites were divided into three main categories, based on the metastatic pathway: intraperitoneal dissemination, lymphatic metastasis, and hematogenous metastasis. In the omental metastasis-positive group, 34 (94.4%) patients had intraperitoneal dissemination, whereas in the omental metastasis-negative group, 13 patients (65.0%) had intraperitoneal dissemination. Omental metastasis was significantly associated with increased intraperitoneal dissemination (*p* = 0.002). Lymphatic metastasis was observed in 17 (47.2%) patients in the omental metastasis-positive group and observed in 14 (70.0%) patients in the metastasis-negative group (*p* = 0.38). In addition, six (16.7%) patients had hematogenous metastasis in the omental metastasis-positive group, whereas one (5.0%) patient had hematogenous metastasis in the metastasis-negative group (*p* = 0.40). Importantly, six (85.7%) of seven patients with hematogenous metastasis also had omental metastasis. Even though no significant difference was observed, these findings suggested that omental metastasis is prone to correlate with increased metastasis to parenchymal organs via the hematogenous route (Table 2).

**Table 2:** Distribution of metastatic sites in eligible patients with stage III–IV ovarian cancer at the time of primary debulking surgery, based on the existence of omental metastasis

Metastatic site	Total (*n* = 56)	Omental metastasis		*p* value
		Positive (*n* = 36)	Negative (*n* = 20)	
Intraperitoneal dissemination, *n* (%)	51 (91.1)	35 (97.2)	13 (65.0)	**0.002**
Mesentery	25 (44.6)	18 (50.0)	7 (35.0)	0.40
Diaphragm	21 (37.5)	18 (50.0)	3 (15.0)	**0.011**
Hepatic surface	20 (35.7)	15 (41.7)	5 (25.0)	0.26
Vesicouterine pouch	9 (16.1)	18 (50.0)	2 (10.0)	**0.009**
Douglas’ pouch	45 (80.4)	34 (94.4)	8 (40.0)	** < 0.001**
Lymphatic metastasis, *n* (%)	31 (55.4)	17 (47.2)	14 (70.0)	0.38
Pelvic lymph nodes	26 (46.4)	14 (38.9)	12 (60.0)	0.17
Para-aortic lymph nodes	18 (32.1)	12 (33.3)	6 (30.0)	1.00
Extra-abdominal lymph nodes	8 (14.3)	6 (16.6)	2 (10.0)	0.70
Hematogenous metastasis, *n* (%)	7 (12.5)	6 (16.7)	1 (5.0)	0.40
Liver	4 (7.1)	4 (11.1)	0 (0.0)	0.29
Bone	2 (3.6)	2 (5.6)	0 (0.0)	0.53
Spleen	2 (3.6)	2 (5.6)	0 (0.0)	0.53
Lung	1 (1.8)	0 (0.0)	1 (5.0)	0.36

Bold values indicate statistically significant *p* values (*p* < 0.05)

### Kaplan–Meier analysis of OS, PFS, and PRS

**Fig. 2:**
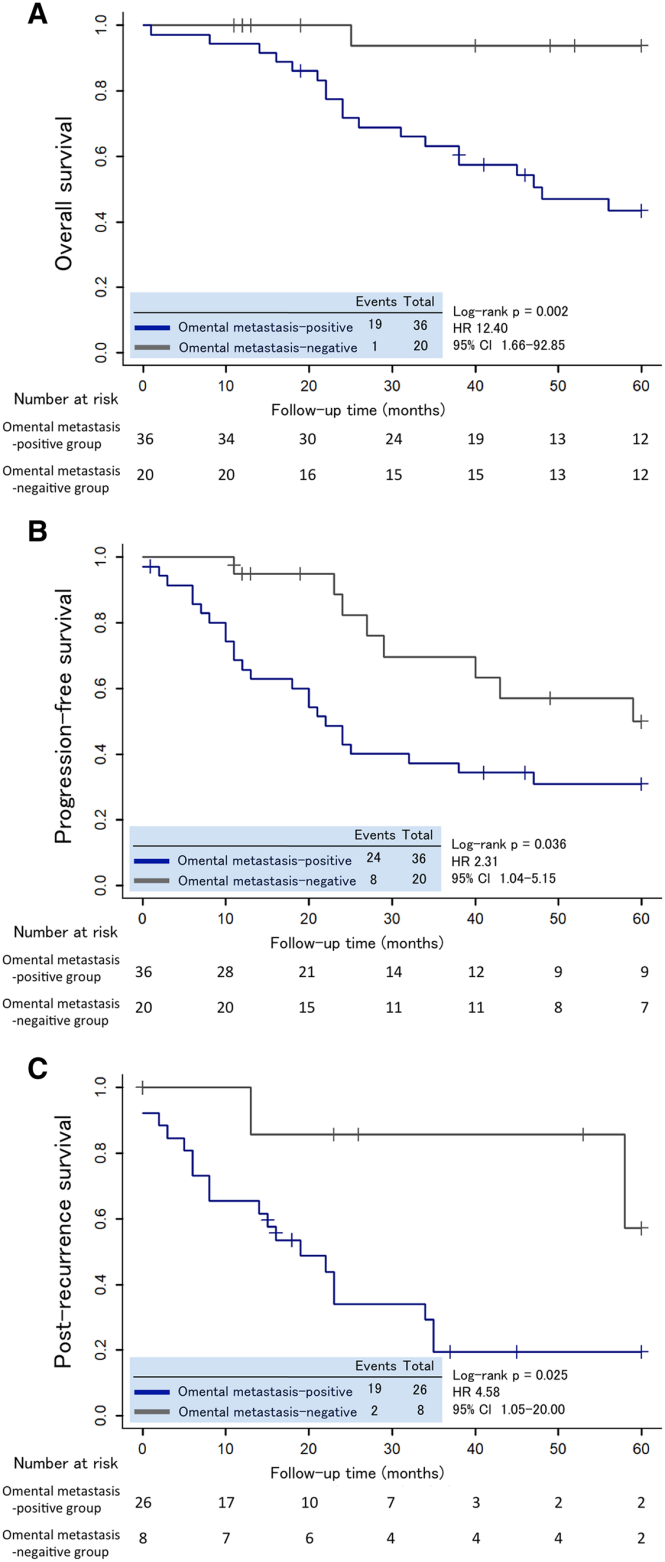
Kaplan–Meier analysis of overall survival (**a**), progression-free survival (**b**), and post-recurrence survival (**c**) of eligible patients with stage III–IV ovarian cancer, based on the existence of omental metastasis. *HR* hazard ratio, *CI* confidence interval

To evaluate whether omental metastasis is associated with survival outcomes in eligible patients with stage III–IV ovarian cancer, we used Kaplan–Meier analysis of OS, PFS, and PRS between the omental metastasis-positive and metastasis-negative groups. The 5-year OS and PFS rates were 43.4% and 30.9%, respectively, in the omental metastasis-positive group, and 93.8% and 49.7%, respectively, in the omental metastasis-negative group. The OS was significantly different between the two groups (HR 12.4; 95% CI 1.66–92.85; *p* = 0.002) (Fig. 2a). Moreover, PFS was significantly shorter in the omental metastasis-positive group than in the metastasis-negative group (HR 2.31; 95% CI 1.04–5.15; *p* = 0.038) (Fig. 2b). In this study, 26 (72.2%) of 36 patients in the omental metastasis-positive group and eight (40.0%) of 20 patients in the omental metastasis-negative group experienced disease recurrence. The recurrence rate between the two groups was significantly different (*p* = 0.024). Furthermore, a comparison of Kaplan–Meier curves for PRS revealed that survival after recurrence was shorter for patients in the omental metastasis-positive group than for patients in the omental metastasis-negative group (HR 4.58; 95% CI 1.05–20.00; *p* = 0.025) (Fig. 2c). These data indicated that omental metastasis is intimately correlated with an unfavorable prognosis in patients with stage III–IV ovarian cancer.

### Univariate and multivariate analysis for OS, PFS, and PRS

To investigate factors that influence the prognosis of eligible patients with stage III–IV ovarian cancer, univariate and multivariate analyses were conducted to identify clinicopathologic factors for OS. Omental metastasis was consequently identified as a predictor of OS, based on the univariate Cox proportional hazards model (HR 12.40; 95% CI 1.66–92.85; *p* = 0.014) and the multivariate Cox proportional hazards model (HR 8.70; 95% CI 1.14–66.69; *p* = 0.037). Our data demonstrated that omental metastasis is an independent risk factor for OS in patients with stage III–IV ovarian cancer (Table 3). With regard to PFS, omental metastasis, FIGO stage, and residual tumor size were statistically associated with poor prognosis in the univariate analysis, whereas the multivariable analysis showed that only residual tumor size was an independent prognostic factor (HR 2.64; 95% CI 1.14–6.11; *p* = 0.024) (Table S1). On another front, only the existence of omental metastasis was significantly related to PRS in the univariate analysis (HR 4.70; 95% CI 1.08–20.48; *p* = 0.039) (Table S2); therefore, multivariate analysis was not required. These results indicated that the existence of omental metastasis served as a prognostic indicator in patients with stage III–IV ovarian cancer.

**Table 3:** Hazard ratios, based on the univariate and multivariate Cox proportional hazard models, for overall survival in eligible patients with stage III–IV ovarian cancer

Variables	Overall survival (OS)			
	Univariate analysis		Multivariate analysis	
	HR (95% CI)	*p* value	HR (95% CI)	*p* value
Age, years				
< 50	Referent			
≥ 50	2.27 (0.82–6.27)	0.11		
BMI, kg/m^2^				
< 18.5	0.33 (0.04–2.71)	0.30		
≥ 18.5 and < 25	0.51 (0.19–1.35)	0.18		
≥ 25	Referent			
Histological type				
High-grade serous	Referent			
Others	1.28 (0.51–3.23)	0.60		
Primary site				
Ovary	Referent			
Tube	0.27 (0.04–2.05)	0.21		
FIGO stage				
III	Referent			
IV	2.05 (0.84–4.98)	0.11		
CA125, U/mL				
< 500	Referent			
≥ 500	0.71 (0.30–1.72)	0.45		
Ascites, mL				
< 500	Referent		Referent	
≥ 500	3.00 (1.24–7.25)	**0.015**	1.70 (0.65–4.46)	0.28
Tumor size, cm				
< 10	Referent			
≥ 10	1.27 (0.53–3.07)	0.59		
Residual tumor size				
Optimal surgery	Referent		Referent	
Suboptimal surgery	3.61 (1.45–9.01)	**0.006**	2.38 (0.89–6.37)	0.09
Omental metastasis				
Negative	Referent		Referent	
Positive	12.4 (1.66–92.85)	**0.014**	9.50 (1.25–72.36)	**0.030**

*BMI* body mass index, *FIGO* International Federation of Gynecology and Obstetrics, *HR* hazard ratio, *CI* confidence interval

Bold values indicate statistically significant *p* values (*p* < 0.05)

### Chemotherapeutic response in patients with and without omental metastasis

Omental metastasis is correlated with poor PRS; therefore, we evaluated the therapeutic effect of chemotherapy for recurrent disease between the omental metastasis-positive and metastasis-negative groups. We analyzed 26 eligible patients with a recurrence of stage III–IV ovarian cancer who underwent chemotherapy for recurrent tumor; 19 patients were included in the omental metastasis-positive group, and seven patients were included in the omental metastasis-negative group. The correlation between omental metastasis and response to chemotherapy is shown in Table 4. CR was achieved in three (15.8%) patients in the omental metastasis-positive group and in five (71.4%) patients in the omental metastasis-negative group. The responses to chemotherapy between the two groups were significantly different (*p* = 0.006). Furthermore, the overall response rate (i.e., CR and PR) to chemotherapy for recurrent tumors was significantly lower in the omental metastasis-positive group than in the omental metastasis-negative group (31.6% vs. 85.7%, *p* = 0.014) (Table 4). Our data indicated that omental metastasis is a risk factor for tumor resistance to chemotherapy in patients with stage III–IV ovarian cancer, suggesting that omental metastasis is intimately correlated with enhanced chemoresistance and consequently has a significant effect on the survival of patients with advanced ovarian cancer.

**Table 4:** Response to chemotherapy for recurrent disease in eligible patients with stage III–IV ovarian cancer, based on the existence of omental metastasis

Response	Total (*n* = 26)	Omental metastasis		*p* value
		Positive (*n* = 19)	Negative (*n* = 7)	
Complete response, *n* (%)	8 (30.8)	3 (15.8)	5 (71.4)	**0.006**
Partial response, *n* (%)	4 (15.4)	3 (15.8)	1 (14.3)	0.93
Stable disease, *n* (%)	4 (15.4)	4 (21.1)	0 (0.0)	0.22
Progressive disease, *n* (%)	10 (38.5)	9 (47.4)	1 (14.3)	0.12
Overall response rate, *n* (%)	12 (46.2)	6 (31.6)	6 (85.7)	**0.014**

Bold values indicate statistically significant *p* values (*p* < 0.05)

## Discussion

The omentum is a large fold of the visceral peritoneum covering the intestine anteriorly in the abdominal cavity and is connected to the colon, spleen, stomach, and pancreas [16]. The omentum has an immunological function in defending the abdominal cavity owing to its ability to attenuate peritoneal inflammation. In addition, the omentum has extraordinary fibrotic and angiogenic activities, which together promote wound healing and neoangiogenesis [17, 18]. In ovarian cancer patients, these distinct activities are involved in the development of omental metastatic tumors [13]. During ovarian cancer metastatic spread, the omentum is one of the most preferred sites of metastasis and frequently forms a large mass that is called an “omental cake.” Of note, a previous study indicated that 43 (76.8%) of 56 patients had omental metastasis in autopsy data of ovarian cancer patients [7]. Omental metastasis is a common phenomenon in ovarian cancer; however, the biological features and clinical significance of omental metastasis remain poorly understood.

In the present study, we aimed to elucidate the effect of omental metastasis on the clinicopathological features and clinical outcomes of patients with stage III–IV ovarian cancer. As a result, significant differences were observed in survival outcomes between the omental metastasis-positive and metastasis-negative groups in stage III–IV ovarian cancer patients. Kaplan–Meier analysis showed that OS, PFS, and PRS were significantly shorter in the omental metastasis-positive group than in the metastasis-negative group. Remarkably, univariate and multivariate analyses demonstrated that omental metastasis is an independent risk factor for the shortened OS. These findings indicated that the existence of omental metastasis is a predictive clinical biomarker for unfavorable survival outcomes in patients with advanced ovarian cancer.

With regard to the metastatic pathway, it has been assumed that ovarian cancer cells preferentially metastasize to the omentum via direct dissemination instead of via hematogenous routes because of the lack of anatomical barriers around the primary lesion of ovarian cancer in the abdominal cavity. However, a recent seminal paper by Pradeep et al. demonstrated a novel paradigm in which ovarian cancer cells metastasize hematogenously with a strong predilection for the omentum [19]. The authors used a parabiosis mouse model that allowed the sharing of blood circulation. They showed that circulating ovarian cancer cells derived from the host mouse first metastasized to the omentum of the conjoined guest mice via a hematogenous route, followed by peritoneal dissemination in the guest mouse. In this regard, we evaluated the association between omental metastasis and other metastatic routes at the time of primary debulking surgery. We found that most patients with hematogenous metastasis also had omental metastasis, raising the possibility that omental metastasis is potentially correlated with increased metastasis to parenchymal organs via a hematogenous route in ovarian cancer patients.

To investigate the causal relationship between omental metastasis and poor prognosis, we evaluated the response to chemotherapy in stage III–IV ovarian cancer patients. Remarkably, our data revealed that patients with omental metastasis were associated with increased chemoresistance for recurrent disease. In a recent clinical report, Böhm et al. demonstrated that the omentum is the prognostically relevant disease site for chemotherapy response assessment [15]. The authors developed a histopathologic scoring system, called the three-tier chemotherapy response score (CRS) system, for measuring the response to neoadjuvant chemotherapy in interval debulking surgery for advanced ovarian cancer. The CRS system showed the prognostic stratification of ovarian cancer patients when applied to omental metastatic disease but not to ovarian tumors. To date, researchers have demonstrated that the omentum is a central player in creating a metastatic tumor microenvironment in the abdominal cavity [20, 21], and various stromal components, such as adipocytes [22–28], mesenchymal stem cells [29, 30], fibroblasts [31, 32], and macrophages [33, 34] in the omental tumor microenvironment enhance the ability to resist chemotherapy in ovarian cancer cells. Previous basic research and our findings further support the theory that metastatic ovarian cancer cells acquire chemoresistance by a reciprocal interaction with stromal cells in the omentum, which subsequently leads to unfavorable survival outcomes. In the current clinical guidelines, omentectomy is included in the standard surgical procedure for all cases of ovarian cancer for assessing adequate surgical staging [10–13]. However, it has been not clear whether excising the omentum has therapeutic significance, despite the recommendation for undergoing omentectomy [14]. Based on the results of our study, we believe that omentectomy effectively may improve a patient’s prognosis by destroying the pathological crosstalk between ovarian cancer cells and stromal components in the omental tumor microenvironment.

The limitations of this study include its retrospective study design at a single institution, potentially causing selection biases. To eliminate these biases, prospective multi-institutional studies need to be conducted. Furthermore, because of making an accurate histopathological diagnosis of omental metastasis, we excluded the patients with stage III–IV ovarian cancer who received neoadjuvant chemotherapy or exploratory laparotomy, leading to a possible selection bias.

In conclusion, our findings demonstrated that omental metastasis is an independent prognostic factor and is associated with increased chemoresistance in stage III–IV ovarian cancer patients. Further comprehensive basic and clinical studies are required to clarify the biological mechanisms and clinical relevance of omental metastasis. Insightful observation and rethinking of distinctive pattern of metastatic spread will be a clue to develop innovative strategies for the diagnosis and treatment of patients with advanced ovarian cancer.

## Supplementary Information

Below is the link to the electronic supplementary material.

Supplementary file1 (DOCX 29 KB)
