# Supplementary material for: Omental metastasis as a predictive risk factor for unfavorable prognosis in patients with stage III–IV epithelial ovarian cancer
Source: Int J Clin Oncol. 2021 Jan 29;26(5):995–1004. doi: 10.1007/s10147-021-01866-3 (PMC8055622; doi:10.1007/s10147-021-01866-3)
Supplement: Supplementary file 1 — Supplementary file1 (DOCX 29 KB) [file 10147_2021_1866_MOESM1_ESM.docx]

# Supplementary Material

**Article title:** Omental metastasis as a predictive risk factor for unfavorable prognosis in patients with stage III-IV epithelial ovarian cancer

**Journal name:** *International Journal of Clinical Oncology*

**Author names:** Yutaka Iwagoi, M.D. ^1^, Takeshi Motohara, M.D., Ph.D. ^1*^, Sangyoon Hwang ^1^, Koichi Fujimoto ^2^, Tokunori Ikeda, M.D., Ph.D. ^3,4^, Hidetaka Katabuchi, M.D., Ph.D. ^1^

**Author affiliation:**

^1^ Department of Obstetrics and Gynecology, Faculty of Life Sciences, Kumamoto University, 1-1-1 Honjo, Chuo-ku, Kumamoto-City, Kumamoto, 860-8556, Japan

^2^ Department of Clinical Laboratory, Fukuoka University Hospital, 7-45-1, Nanakuma, Jonan-ku, Fukuoka-City, Fukuoka, 814-0180, Japan

^3^ Laboratory of Clinical Pharmacology and Therapeutics, Faculty of Pharmaceutical Sciences, Sojo University, 4-22-1, Ikeda, Nishi-ku, Kumamoto-City, Kumamoto, 860-0082, Japan

^4^ Department of Medical Information Sciences and Administration Planning, Kumamoto University Hospital, 1-1-1 Honjo, Chuo-ku, Kumamoto-City, Kumamoto, 860-8556, Japan

**Corresponding author:** Takeshi Motohara

Fax: +81-96-363-5164　 Tel: +81-96-373-5269

E-mail: kan@kumamoto-u.ac.jp

Table S1. Hazard ratios, based on the univariate and multivariate Cox proportional hazard models, for progression-free survival in eligible patients with stage III-IV ovarian cancer

| Variables | **Progression-free survival (PFS)** | | | |
| --- | --- | --- | --- | --- |
|  | Univariate analysis | | Multivariate analysis | |
|  | HR (95% CI) | p value | HR (95% CI) | p value |
| Age, years |  |  |  |  |
| <50 | Referent |  |  |  |
| ≥50 | 1.54 (0.74-3.20) | 0.25 |  |  |
| BMI, kg/m^2^ |  |  |  |  |
| <18.5 | 0.48 (0.10-2.28) | 0.36 |  |  |
| ≥18.5 and <25 | 0.70 (0.31-1.59) | 0.40 |  |  |
| ≥25 | Referent |  |  |  |
| Histological type |  |  |  |  |
| High-grade serous | Referent |  |  |  |
| Others | 0.97 (0.45-2.10) | 0.94 |  |  |
| Primary site |  |  |  |  |
| Ovary | Referent |  |  |  |
| Tube | 0.43 (0.13-1.42) | 0.17 |  |  |
| FIGO stage |  |  |  |  |
| III | Referent |  |  |  |
| IV | 2.95 (1.46-5.97) | **0.003** | 1.84 (0.82-4.11) | 0.14 |
| CA125, U/mL |  |  |  |  |
| <500 | Referent |  |  |  |
| ≥500 | 1.29 (0.63-2.63) | 0.49 |  |  |
| Ascites, mL |  |  |  |  |
| <500 | Referent |  |  |  |
| ≥500 | 2.20 (1.08-4.45) | 0.030 | 1.26 (0.57-2.80) | 0.57 |
| Tumor size, cm |  |  |  |  |
| <10 | Referent |  |  |  |
| ≥10 | 1.20 (0.60-2.39) | 0.62 |  |  |
| Residual tumor size |  |  |  |  |
| Optimal surgery | Referent |  | Referent |  |
| Suboptimal surgery | 3.92 (1.86-8.26) | **<0.001** | 2.64 (1.14-6.11) | **0.024** |
| Omental metastasis |  |  |  |  |
| Negative | Referent |  | Referent |  |
| Positive | 2.31 (1.04-5.15) | **0.041** | 2.00 (0.89-4.51) | 0.09 |

BMI, body mass index; FIGO, International Federation of Gynecology and Obstetrics;

HR, hazard ratio; CI, confidence interval.

Table S2. Hazard ratios, based on the univariate Cox proportional hazard models, for post-recurrence survival in eligible patients with stage III-IV ovarian cancer

| Variables | **Post-recurrence survival (PRS)** | |
| --- | --- | --- |
|  | Univariate analysis | |
|  | HR (95% CI) | p value |
| Age, years |  |  |
| <50 | Referent |  |
| ≥50 | 2.42 (0.88-6.67) | 0.09 |
| BMI, kg/m^2^ |  |  |
| <18.5 | 0.65 (0.77-5.47) | 0.69 |
| ≥18.5 and <25 | 0.44 (0.16-1.21) | 0.11 |
| ≥25 | Referent |  |
| Histological type |  |  |
| High-grade serous | Referent |  |
| Others | 1.25 (0.50-3.11) | 0.63 |
| Primary site |  |  |
| Ovary | Referent |  |
| Tube | 1.20 (0.16-9.30) | 0.86 |
| FIGO stage |  |  |
| III | Referent |  |
| IV | 1.16 (0.47-2.83) | 0.75 |
| CA125, U/mL |  |  |
| <500 | Referent |  |
| ≥500 | 0.73 (0.31-1.74) | 0.48 |
| Ascites, mL |  |  |
| <500 | Referent |  |
| ≥500 | 2.00 (0.84-4.72) | 0.12 |
| Tumor size, cm |  |  |
| <10 | Referent |  |
| ≥10 | 1.39 (0.59-3.27) | 0.46 |
| Residual tumor size |  |  |
| Optimal surgery | Referent |  |
| Suboptimal surgery | 1.61 (0.67-3.88) | 0.29 |
| Omental metastasis |  |  |
| Negative | Referent |  |
| Positive | 4.70 (1.08-20.48) | **0.039** |

BMI, body mass index; FIGO, International Federation of Gynecology and Obstetrics;

HR, hazard ratio; CI, confidence interval.
